# Diarrhea Prevalence and Sociodemographic Factors among Under-Five Children in Rural Areas of North Gondar Zone, Northwest Ethiopia

**DOI:** 10.1155/2018/6031594

**Published:** 2018-06-03

**Authors:** Atalay Getachew, Tadesse Guadu, Alebachew Tadie, Zemichael Gizaw, Mulat Gebrehiwot, Daniel Haile Cherkos, Martha Alemayehu Menberu, Teklay Gebrecherkos

**Affiliations:** ^1^University of Gondar, College of Medicine and Health Sciences, Institute of Public Health, Department of Environmental & Occupational Health & Safety, P.O. Box 196, Gondar, Ethiopia; ^2^University of Gondar, College of Natural and Computational Sciences, Department of Biology, P.O. Box 196, Gondar, Ethiopia; ^3^University of Gondar, College of Medicine and Health Sciences, School of Biomedical and Laboratory Sciences, Department of Medical Microbiology, P.O. Box 196, Gondar, Ethiopia

## Abstract

**Background:**

Diarrheal disease remains one of the principal causes of morbidity and mortality in infants and children in developing countries, including Ethiopia. Risk factors for diarrhea vary by settings and have important implications for developing intervention strategies to reduce the burden of the disease. Thus, the aim of this study was to assess diarrhea prevalence and sociodemographic factors among under-five children in rural areas of North Gondar Zone.

**Methods:**

A community-based cross-sectional study was conducted from April to June 2016 among 736 randomly selected households with one child under five years old. A structured questionnaire was used for collecting information on sociodemographic characteristics and diarrheal occurrence. Data was analyzed using SPSS version 20. The bivariate and multivariable logistic regression analysis were used to determine the association between risk factors and diarrheal occurrence, and a p value < 0.05 was taken as statistically significant.

**Results:**

A total of 736 under-five children and their respondents were enrolled during the study period. Almost all respondents were biological mothers 96.4% (709/736), married 94.2% (693/736), and house wives 86% (632/736). The overall prevalence of diarrheal disease among under-five children was 22.1% (163/743). Of these, children with age group of less than one year old, 7.7 % (57/736), were commonly infected with diarrheal diseases. Children less than or equal to one year [AOR=1.82, 95% CI= (1.39, 4.63)], guardians [AOR=4.37, 95% CI= (1.73, 11.1)], and children with no breast feeding practice [AOR=3.13, 95% CI= (1.62, 6.03)] were the major risk factors for the occurrence of diarrhea.

**Conclusion:**

Childhood diarrhea remains an important health concern in the study area. Occurrence of diarrhea was statistically associated with child age less than or equal to one year, educational status of mother/guardians, and breast feeding. To minimize the magnitude childhood diarrhea, various designing and implementing strategies, such as health education, child care, breast feeding, and weaning practice, integrated with the existing national health extension are quite essential.

## 1. Introduction

Diarrhea is one of the waterborne diseases which are reported as the leading cause of death in infants and children [1]. According to WHO, diarrhea is the passage of 3 or more times loose or liquid stools per day or more frequently than the normal for the individual. Globally, diarrheal disease remains one of the principal causes of morbidity and mortality in children. In the globe, under-five children experience on an average 3.2 episodes of diarrhea every year [2] and consequently 1.87 million children will die from dehydration associated with diarrheal disease [3].

In the developing countries, diarrheal disease among under-five children accounts for about 21% of all deaths [4, 5]. Of this, it is estimated that 15-20% or more percentage of community diarrheal disease is caused by unsafe drinking water [6]. Ethiopian child mortality rate in 2007 was 199 per 1,000 births, and approximately one of every five deaths every year is due to diarrheal disease [3]. Diarrhea is one of the waterborne diseases which is reported as the leading cause of death in under-five children [7]. Very unfortunately, 46% of under-five child mortality is due to diarrhea in which water related diseases take the higher percentage.

There are different factors associated with occurrence of diarrhea among children less than five years and these differ from place to place. A study carried out in Kenya showed that six factors were independently associated with diarrheal diseases, occupation of the parent/guardian, care taker not washing hands after changing napkins, child drinking untreated water from the river, child not exclusively breastfed, child not washing hands before eating and after visiting toilet [8].

In Ethiopia, several interventions are going on to reduce morbidity and mortality of children; consistent water source protection is the first line of defense against diarrheal diseases and it is the best method of ensuring safe drinking water [9]. In addition, household level water quality interventions can significantly reduce diarrheal diseases that are caused by pathogens [10]. To do so, regular and frequent assessment of the bacteriological quality of water is needed to get information about diarrheal diseases so as to apply sustainable monitoring system to control the water quality status of municipal and rural water distribution systems [11].

However, in North Gondar Zone, no study is available on the prevalence of diarrhea in under-five children. Therefore, this study aims to assess the prevalence of diarrheal disease and associated sociodemographic factors among under-five children in North Gondar Zone rural areas and intervene improvement strategies based on the assessment result.

## 2. Materials and Methods

### 2.1. Study Area

This study was conducted in North Gondar Zone from April to June 2016. Gondar is located 739 km far from Addis Ababa to the northwest of Ethiopia. North Gondar is one of the eleven zones in Amhara Regional State. It has 22 administrative woredas (districts). As per the data gained from North Gondar Zonal Health Department, the total projected population in 2015/16 was 3,704,740. The majority of which, 2920007 (78.8%), reside in rural areas whereas the rest, 784733 (21.2%), were in urban areas. Moreover, 499542 (13.5%) of the population were under-five children. According to the reports of the North Gondar Zonal Health Department (2014/15), diarrheal diseases are one of the top ten diseases in the North Gondar Zone.

### 2.2. Study Design and Period

A community-based cross-sectional study design was employed to collect data from households to assess diarrhea prevalence and associated factors from April to June 2016.

### 2.3. Source Population

All households were in North Gondar Zone with mothers/guardians having under-five children during the study period.

### 2.4. Study Population

All households were in North Gondar Zone with mothers/guardians having under-five children with diarrhea.

### 2.5. Inclusion Criteria

All mothers/guardians who have under-five children with diarrhea at selected households were included in the study.

### 2.6. Exclusion Criteria

(i) Children who were chronically ill and with persistent diarrhea for greater than two weeks were excluded.

(ii) Critically ill or suffering mothers or guardians of the index child were excluded, since it is unethical to take routine information from a suffering person or difficult to obtain complete information.

### 2.7. Variables of the Study

#### 2.7.1. Dependent Variable

The dependent variable is diarrheal disease prevalence.

#### 2.7.2. Independent Variables

The independent variables are sociodemographic characteristics (age, sex, address, educational status, occupation, family members), household size, parental education, maternal occupation, family size, maternal age, number of children under-five years of age, child's age, vaccination status, and breast feeding practice.

### 2.8. Sample Size and Sampling Procedure

#### 2.8.1. Magnitude of Diarrhea

Diarrhea is the passage of 3 or more times loose or liquid stools per day or more frequently than the normal for the individual [2]. The sample size was determined by using the single population proportion formula based on an assumption that 18% of the under-five children had two-week prevalence of diarrhea in North Gondar [12], with marginal error of 4 %, a standard score corresponding to 95% certainty, design effect of 2, accounting for two-stage sampling and nonresponse rate 5%. The total sample size included in the study was 743 households that had at least one under-five child. Four districts (Dembia, Gondar Zuria, Chilga, and Sanja) were randomly selected among the 22 total districts of North Gondar Zone, and multistaged sampling procedure was employed.

Using probability proportional (PPS) to size, the number of households was determined in each district. Then, 25% of total kebeles were selected from each district by simple random sampling technique, and systematic sampling technique was applied to select study households. In case there are more than one under-five child in the same household, index child was selected by lottery method to collect information on child's health characteristics. The first household interview was identified by a modified random walk method, and if there is no mother/guardians or under-five child in the selected household, the next nearest household was included in the survey.

#### 2.8.2. Data Collection Tool and Method

The data collection tool was structured interview questionnaire to be filled by data collector among randomly selected household to estimate the magnitude of diarrhea in under-five children.

#### 2.8.3. Data Management and Quality Control

The questionnaire was prepared originally in English and translated into Amharic and back to English to keep the consistency of the questions by independent individuals. Training of the data collection team was made to insure the possible quality data. The principal investigator and supervisors checked and reviewed the filled questionnaires to ensure completeness and consistency of the information collected. Incorrectly filled or missed questionnaires were turned back to the data collector for correction in the next day.

### 2.9. Data Management and Analysis

All collected data was checked for completeness and reliability before entry into software. Data entry and cleaning was done using Epi Info version 3.5.3 computer software. Descriptive statistics of SPSS version 20 was used to summarize all the values of the variables. Pearson's x^2^ test and binary logistic regression with 95 % CI were computed as measures of association. To assess the association between the different predictor variables of diarrheal occurrence in under-five children with the dependent variables, first binary relationships between each independent variable and outcomes were investigated using a binary logistic regression model. All variables with P value less than 0.2 were included in the multiple logistic regression models, and P value of less than 0.05 was considered as statistically significant.

### 2.10. Ethical Approval

Ethical clearance was obtained from the Ethical Committee of the University of Gondar. Permission and support letter was obtained from North Gondar Zone Health Bureaus. The purpose and importance of the study was explained to each study participant. To ensure confidentiality of participants' information, anonymous typing was applied whereby the name of the participant and any identifier of participants were not written on the questionnaire, and to keep the privacy during the interview, they were interviewed alone. Above all data was collected after full verbal consent was obtained from study participants. Children who have diarrhea during the interview were treated using ORS and zinc tablets and then sent to nearby hospital.

## 3. Results

### 3.1. Sociodemographic Characteristics and Occurrence of Diarrheal Disease

A total of 736 under-five children and their mothers with 99% (736/743) response rate from different rural areas of North Gondar Zone (Dembia, Gondar Zuria, Chilga, and Sanja) were enrolled during the study. Of these more than half, 55.7% (410/736), were males. Majority of mothers were in the age group of 25-34 years with mean age of 30.7%. Almost all respondents were biological mothers 96.4% (709/736), married 94.2% (693/736), and house wives 86% (632/736). The educational status of mothers/guardians showed that 58% (431/736) of them were unable to read and write, while only 4.5% of them attended high school and above. The overall prevalence of diarrheal disease among under-five children was 22.1% (163/736). Of these, children with age group of less than one year old, 7.7 % (57/736), were commonly infected with diarrheal diseases; moreover, children who practice partial breast feeding were more infected with diarrheal disease, 15.5 (114/736), while 2.5%(18/736) of them who practice exclusive breastfeeding were less infected with diarrheal diseases. Households with family size of less than five were reported to be highly exposed to diarrheal diseases, 14.3% (105/736) (Table 1).

### 3.2. Factors Associated with Diarrheal Diseases among Under-Five Children

Bivariate and multivariate analysis on socioeconomic variables, like the age, district hygiene and sanitary condition, educational status of father, and current breast feeding, are shown in Table 2. The occurrence of diarrheal disease was associated with the number and age of under-five children in the households. The occurrence of diarrhea was 1.82 times more likely to be higher among households with less than or equal to one-year children compared with households with four- to five-year children [AOR=3.89, 95% CI= (1.58, 9.57)]. The occurrence of diarrhea was significantly associated with district hygiene and sanitary condition. In relation to hygiene and sanitary condition, the probability of diarrheal occurrence among under-five children was 3 times higher in Dembia district compared to Sanja district. Childhood diarrheal disease was statistically associated with the educational status of mothers/caretakers. The likelihood of diarrheal occurrence was 4.37 times higher among children whose mothers/guardians had unable to read compared with educational status of grade >12 and above [AOR=4.37, 95% CI= (1.73, 11.1)].

Moreover, current breast feeding practice was significantly associated with occurrence of diarrheal disease. The risk of developing diarrhea was 3.13 times higher among children whose mothers had no breast feeding practice [AOR=3.13, 95% CI= (1.62, 6.03)] compared to children whose mothers had exclusive feeding.

## 4. Discussion

This study investigated the prevalence of diarrheal occurrence and sociodemographic characteristics among under-five children in rural areas of North Gondar Zone. The overall prevalence of diarrhea in this study was 22.1%; this was in line with the study conducted in eastern Ethiopia, 22.5% [13, 14], and Somalia region. However, it was lower than those reported in the studies conducted in Burundi rural areas, 32.6% [15]; nomadic population in Afar region, 26.1% [16]; Arba Minch, Southern Nationalities, and Peoples' region, 30.5% [17]; and Jijiga, Somalia region 27.3%[14]. This might be due to the inclusion of only rural children and the difference in provision health service between rural and urban population. However the current finding was higher than the finding of the Ethiopian demographic and health survey 2016 (EDHS), in which the magnitude of diarrheal disease among children younger than 5 years old was 12% [18], study conducted in West Gojjam, 18% [19], and North Gondar Zone, Amhara region 15% [20]. The possible explanation for this difference could be the variation in the sociodemographic characteristics of the study subjects, socioeconomic development, and study periods. People's living style, behavioral change, and communication strategies of these areas could also contribute notably to such variations.

The children aged 0-1 year old were at high risk of developing diarrhea compared to children aged b/n 4-5 years old. This findings was in line with studies conducted in Arba Minch, southern Ethiopia [17], India [21], Sudan [22], and Thailand [23]. The high prevalence of diarrhea at this age could be due to the low immunity of children, introduction of contaminated weaning foods, and crawling starting at this age and the risk of ingesting contaminated foods and drinks.

The present study found that the odds of diarrheal diseases of children whose mothers/caretakers cannot read and write were higher than those of children whose mothers/guardians had grade greater than 12 and above level of education. This is also similar to previous studies conducted in Jijiga, Somalia region [14]; Hadaleala district, Afar region, northeast Ethiopia [16]; Arba Minch, southern Ethiopia [17]; and Sheko district, southwest Ethiopia [24]. This may be due to the fact that education is likely to enhance household health and sanitation practices. Education can increase awareness about the transmission and prevention methods of diarrhea. It also encourages changes in behavior at the household level.

Findings of our study showed that diarrheal occurrence was associated with children that do not have breast feeding in their early age. This was also agreed with other reports from Gojjam, west Ethiopia, indicating that not breastfeeding resulted in an excess risk of diarrhea mortality in comparison to exclusive breastfeeding among infants aged 0-5 months and to any breastfeeding among children aged 6-23 months [25, 26].

## 5. Conclusion

The study revealed that childhood diarrhea remains an important health concern in the study area. The highest rate of the occurrence of diarrhea was significantly seen among children aged 0-1 year old. Occurrence of diarrhea was statistically associated with child age of less than or equal to one, educational status of mother/guardians and breast feeding. To minimize the magnitude childhood diarrhea, various designing and implementing strategies, such as health education, child care, early vaccination of children, and weaning practice, integrated with the existing national health extension are quite essential.

### 5.1. Limitation of the Study

Maternal/guardians diarrheal history was not collected.

## Figures and Tables

**Table 1:** Sociodemographic characteristics of respondents among under-five children in rural areas of North Gondar Zone, Northwest Ethiopia, 2016.

**Variables**	**Diarrhea (N** = **736)**		
	**Yes (**%**)**	**No (**%**)**	**P value**
**Age group of index child**			
0-1 year	57 (7.7)	134 (18.2)	0.001*∗*
1-2 years	47 (6.4)	127 (17.3)	
2-3 years	38 (5.2)	138 (18.8)	
3-4 years	15 (2.0)	119 (16.2)	
4-5 years	06 (0.8)	55 (7.5)	
**Sex of index child**			
Male	91 (12.4)	319 (43.3)	0.97
Female	72 (9.8)	254 (34.5)	
**District hygiene and sanitary condition**			
Dembia	64 (8.7)	96 (13.0)	

			0.001*∗*
Gondar Zuria	36 (4.8)	175 (23.8)	
Chilga	22 (3.0)	158 (21.5)	
Sanja	41 (5.6)	144 (19.6)	
**Relation of the respondent to the child**			
Mother	155 (21.1)	554 (75.3)	
Father	05 (0.6)	16 (2.2)	
			0.25
Caretaker	03 (0.4)	03 (0.4)	
Age group of mother/caretaker			
15-24 years	36 (4.9)	115 (15.6)	
			0.55
25-34 years	90 (12.2)	272 (37.0)	
>35 years	37 (5.0)	186 (25.3)	
**Marital status of mother/ guardian**			
Married	150 (20.4)	543 (73.8)	
			0.045*∗*
Divorced	10 (1.4)	25 (3.4)	
Single	02 (0.2)	0 (0)	
Widowed	01 (0.1)	05 (0.7)	
Religion of parents			
Orthodox	159 (21.6)	531 (72.2)	
			0.067
Protestant	01 (0.1)	05 (0.7)	
Muslim	03 (0.4)	37 (5)	
**Educational status of mother/guardian**			
Illiterate	85 (11.6)	346 (47)	

			0.001*∗*
Read and write	18 (2.5)	44 (6.0)	
Grade 1-8	34 (4.6)	101 (13.7)	
Grade 9-12	10 (1.4)	65 (8.8)	
Greater than grade 12	16 (2.2)	17 (2.3)	
Occupation of mother			
Housewife	138 (18.8)	494 (67.1)	
			0.001*∗*
Government employee	12 (1.6)	12 (1.60	
Others (farmer, merchant, etc.)	13 (1.8)	67 (9.1)	
Age of father			
15-24 years	03 (0.4)	12 (1.6)	
			0.034*∗*
25-34 years	79 (10.7)	213 (28.9)	
>35 years	81 (11.0)	348 (47.3)	
**Educational status of father**			
Illiterate	63 (8.8)	291 (40.5)	

			0.002*∗*
Read and write	33 (4.6)	89 (12.4)	
Grade 1-8	32 (4.5)	105 (14.6)	
Grade 9-12	20 (2.8)	71 (9.9)	
Greater than grade 12	08 (1.1)	07 (1.0)	
**Occupation of father**			
Farmer	121 (16.8)	444 (61.8)	
			0.36
Government employee	13 (1.8)	50 (7.0)	
Others (farmer, daily labor, etc.)	22 (3.10)	67 (9.4)	
Family size			
≤ 5	105 (14.3)	358 (48.6)	
			0.65
>5	58 (7.9)	215 (29.2)	
**Average monthly income /ETB/**			
≤ 500	22 (3.0)	95 (12.9)	
			0.75
501-1000	74 (10.1)	232 (31.5)	
>1000	67 (9.1)	246 (33.4)	
**Birth order**			
1^st^	47 (6.4)	214 (29.1)	

			0.66
2^nd^	41 (5.6)	93 (12.6)	
3^rd^	26 (3.5)	98 (13.3)	
4^th^ and above	49 (6.7)	168 (22.8)	
**Current breastfeeding status**			
Exclusive breastfeeding	18 (2.5)	56 (7.6)	
			0.001*∗*
Partial breastfeeding	114 (15.5)	255 (34.6)	
Not breastfeeding	31 (4.2)	262 (35.6)	
**Duration of breastfeeding**			
≤ 1 year	59 (8.0)	141 (19.2)	
			0.34
1-2 years	64 (8.7)	251 (34.1)	
>2 years	40 (5.4)	181 (24.6)	
**Beginning age of supplementary feeding**			
<6 months	11 (1.5)	25 (3.4)	
			0.67
6-12 months	134 (18.2)	481 (65.4)	
>12 months	01 (0.1)	17 (2.3)	
**Measles vaccination status**			
Yes	120 (16.3)	470 (63.9)	0.018*∗*
No	43 (5.8)	103 (14.0)	
**Rota virus vaccination status**			
Yes	109 (14.8)	358 (48.6)	0.30

No	54 (7.3)	215 (29.2)	

ETB: Ethiopian birr, N: number, *∗*P value less than 0.05.

**Table 2:** Bivariate and multivariate analysis of sociodemographic factors related to diarrhea among under-five children in rural areas of North Gondar Zone, Northwest Ethiopia, 2016.

**Variables**	**Diarrhea (N = 736)**		**COR (95**%** CI)**	**AOR (95**%** CI)**
	**Yes (**%**)**	**No (**%**)**		
**Age group of index child**				
0-1 year	57 (7.7)	134 (18.2)	3.899 (1.589-9.570)*∗*	1.824 (1.396-4.163)*∗*
1-2 years	47 (6.4)	127 (17.3)	3.392 (1.370-8.400)*∗*	1.473 (0.484-4.486)
2-3 years	38 (5.2)	138 (18.8)	2.524 (1.010-6.308)*∗*	1.458 (0.518-4.103)
3-4 years	15 (2.0)	119 (16.2)	1.155 (0.425-3.138)	0.681 (0.222-2.085)
4-5 years	06 (0.8)	55 (7.5)	1	1
**District hygiene and sanitary condition**				
Dembia	64 (8.7)	96 (13.0)	2.341 (1.464-3.744)*∗∗*	3.035 (1.674-5.504)*∗*
Gondar Zuria	36 (4.8)	175 (23.8)	0.723 (0.439-1.190)	1.150 (0.636-2.081)
Chilga	22 (3.0)	158 (21.5)	0.489 (0.278-0.860)*∗*	0.822 (0.394-1.714)
Sanja	41 (5.6)	144 (19.6)	1	1
**Relation of the respondent to the child**				
Mother	155 (21.1)	554 (75.3)	1	1
Father	05 (6.8)	16 (2.2)	1.117 (0.403-3.097)	1.949 (0.639-5.948)
Caretaker	03 (0.4)	03 (0.4)	3.574 (0.714-17.884)	6.125 (0.955-9.267)
**Age group of mother/guardian**				
15-24 years	36 (4.9)	115 (15.6)	1.574 (0.941-2.632)	1.166 (0.578-2.355)
25-34 years	90 (12.2)	272 (37.0)	1.663 (1.087-2.546)*∗*	1.577 (0.950-2.620)
>35 years	37 (5.0)	186 (25.3)	1	1
**Religion of parents**				
Orthodox	159 (21.6)	531 (72.2)	3.693 (1.124-12.137)*∗*	1.873 (0.516-6.796)
Protestant	01 (0.1)	05 (0.7)	2.467 (0.213-28.535)	1.096 (0.084-14.386)
Muslim	03 (0.4)	37 (5)	1	1
**Educational status of mother/guardian**				
Unable to read and write	85 (11.6)	346 (47)	4.076 (1.933-8.592)*∗∗*	4.373 (1.725-11.085)*∗*
Read and write	18 (2.5)	44 (6.0)	1.713 (1.056-2.777)*∗*	1.778 (1.024-3.089)*∗*
Grade 1-8	34 (4.6)	101 (13.7)	1.408 (0.871-2.276)	2.110 (1.128-3.947)*∗*
Grade 9-12	10 (1.4)	65 (8.8)	1.301 (0.739-2.292)	2.377 (1.021-5.535)*∗*
Greater than grade 12	16 (2.2)	17 (2.3)	1	1
**Educational status of father **				
Unable to read and write	63 (8.6)	291 (39.5)	3.831 (1.860-7.892)*∗∗*	2.136 (0.810-5.638)
Read and write	33 (4.5)	89 (12.1)	1.665 (0.916-3.027)	1.085 (0.537-2.192)
Grade 1-8	32 (4.4)	105 (14.3)	1.370 (0.869-2.161)	1.131 (0.609-2.102)
Grade 9-12	20 (2.7)	71 (9.6)	0.626 (0.309-1.270)	0.392 (0.154-1.001)
Greater than grade 12	15 (2.0)	17 (2.3)	1	1
**Current breastfeeding status**				
Exclusive breastfeeding	18 (2.5)	56 (7.6)	1	1
Partial breastfeeding	114 (15.5)	255 (34.6)	2.717 (1.420-5.196)*∗*	1.981 (0.717-5.476)
Not breast feeding	31 (4.2)	262 (35.6)	3.778 (2.451-5.825)*∗∗*	3.128 (1.622-6.033)*∗∗*
**Measles vaccination status**				
Yes	120 (16.3)	470 (63.9)	1	1

No	43 (5.8)	103 (14.0)	1.635(1.087-2.460)*∗*	1.190 (0.615-2.303)

**∗**Significant association at p<0.05, *∗∗*Significant association at p<0.001, ^1^Occurrence of child diarrhea.

## Data Availability

Data were collected from each rural area of households having under-five children from North Gondar Administrative Zone, northwest Ethiopia, registered on Microsoft Excel spreadsheet, and entered to SPSS version 20, and all data generated or analyzed during this study are included in this manuscript.

## Abbreviations

AOR: Adjusted odds ratio
COR: Crude odds ratio
Cl: Confidence interval
ETB: Ethiopian birr
N: Number
PPS: Probability proportion to size
WHO: World Health Organization.
